# *NOTCH1* as a Negative Regulator of Avian Adipocyte Differentiation: Implications for Fat Deposition

**DOI:** 10.3390/ani14040585

**Published:** 2024-02-09

**Authors:** Zheng Wang, Yue Su, Mingyu Zhao, Zhenhua Ma, Jianhui Li, Zhuocheng Hou, Huifeng Li

**Affiliations:** 1 College of Life Sciences, Shanxi Agricultural University, Jinzhong 030801, China; wangzheng@sxau.edu.cn (Z.W.); 17303454662@163.com (Y.S.); zmy1275265374@163.com (M.Z.); tianfei0303@163.com (Z.M.); 2 College of Animal Science, Shanxi Agricultural University, Jinzhong 030801, China; jianhui19840717@163.com; 3 National Engineering Laboratory for Animal Breeding and MARA Key Laboratory of Animal Genetics, Breeding and Reproduction, College of Animal Science and Technology, China Agricultural University, Beijing 100193, China; zchou@cau.edu.cn

**Keywords:** avian, adipocyte differentiation, metabolic regulation, *NOTCH1*, *HES1*

## Abstract

**Simple Summary:**

Excessive abdominal fat deposition significantly impacts feed efficiency and meat yield of chicken, which is closely associated with adipocyte differentiation (adipogenesis). Adipogenesis is regulated by various transcription factors and signaling molecules. Our previous transcriptome study found that the NOTCH signaling pathway is significantly enriched during avian adipocyte differentiation. However, in previous studies, *NOTCH1* exerts both positive and negative effects on adipogenesis. Here, we perturbed *NOTCH1* expression in chicken adipocytes using different strategies and investigated the effects of *NOTCH1* on gene expression and adipogenesis. This study demonstrated that *NOTCH1* is a key regulator of avian adipogenesis and provides a potential target for molecular breeding to reduce abdominal fat deposition in chicken.

**Abstract:**

The NOTCH signaling pathway plays a pivotal role in diverse developmental processes, including cell proliferation and differentiation. In this study, we investigated whether this signaling molecules also contribute to avian adipogenesis. Using previous mRNA-seq datasets, we examined the expression of 11 signaling members during avian adipocyte differentiation. We found most members are down-regulated throughout differentiation (*p* < 0.05). As a representative, *NOTCH1* was decreased in cultured chicken abdominal adipocytes during adipogenesis at mRNA and protein levels (*p* < 0.05). Moreover, using an overexpression plasmid for NOTCH1’s intracellular domain (NICD1), as well as siRNA and DAPT to activate or deplete *NOTCH1* in cells, we investigated the role of *NOTCH1* in avian adipogenesis. Our findings illuminate that *NOTCH1* activates the expression of *HES1* and *SOCS3* while it decreases *NR2F2* and *NUMB* (*p* < 0.05), as well as inhibits oleic acid-induced adipocyte differentiation (*p* < 0.01). We further demonstrate that *HES1*, a downstream transcription factor activated by *NOTCH1*, also significantly inhibits adipogenesis by suppressing *PPARγ* and *C/EBPα* (*p* < 0.01). Collectively, these findings establish *NOTCH1* as a negative regulator of avian adipocyte differentiation, unveiling NOTCH signaling as a potential target for regulating avian fat deposition.

## 1. Introduction

Owing to the intensive genetic selection for growth rate and weight, poultry have experienced excessive abdominal fat accumulation, which adversely affects feed efficiency and meat production [1]. Fat tissue functions as a crucial energy storage organ, playing pivotal roles in sustaining the metabolic health of avian species [2]. However, dysfunctional abdominal fat tissue is usually correlated with metabolic disorders, which may lead to fatty liver disease and more mortality [3]. Hence, a thorough investigation into the molecular regulatory mechanisms governing fat development can contribute to the genetic improvement of avian abdominal fat deposition.

The development of adipose tissue results from both an increase in the number of adipocytes (hyperplasia) and the promotion of adipocyte differentiation (hypertrophy) [4]. Excessive fat deposition is closely linked with adipocytes differentiation during animal growth [5]. The process of adipocyte differentiation and lipid accumulation is governed by a meticulously regulated cascade of transcription factors, which includes members of the CCAAT/enhancer binding protein (C/EBP) family (*C/EBPα*, *β*, and *δ*), along with the nuclear receptor peroxisome proliferator activated receptor γ (*PPARγ*) [6,7]. In addition, many signaling pathways, such as WNT [8], have been reported to be involved in regulating adipocyte differentiation. Recently, our group utilized the established chicken abdominal adipocyte model (ICP1 cells) to analyze mRNA expression at different stages of differentiation, and identified the function of *MYOD1* in avian adipogenesis [9]. Moreover, we also explored the differentially expressed genes during duck subcutaneous adipocyte differentiation process through mRNA-seq and mapped an elaborate network of potential transcription factors that regulate differentiation [10]. Notably, NOTCH signaling was enriched significantly in the early regulatory annotation of the differentiation of both types of avian adipocytes.

NOTCH signaling plays a conserved role in connecting cell membrane events with transcriptional regulation, impacting cell fate decisions in multicellular organism development [11]. Four transmembrane NOTCH receptors (*NOTCH1–4*) and the five canonical ligands (*DLL1*, *DLL3*, *DLL4*, *JAGGED1*, and *JAGGED2*), along with the two non-canonical ligands (*DLK1* and *DLK2*), constitute the NOTCH signaling [12]. The NOTCH receptor–ligand interaction leads to the release of NOTCH intracellular domain (NICD) within signal–receiving cells, which then enters the nucleus and interacts with RBP–Jκ to regulate gene expression, including the *HES1* and HES–related family bHLH transcription factor with YRPW motif 1, 2 (*HEY1*, *HEY2*) [13,14]. However, current genome versions of chicken (GRCg7b) and duck (CAU_wild_1.0) lack gene annotations for *DLL3*, *NOTCH3*, and *NOTCH4*. Moreover, recent evidence has found that NOTCH signaling could activate Suppressor of Cytokine Signaling 3 (*SOCS3*) in macrophages [15] and repressed by Nuclear Receptor Subfamily 2, Group F, Member 2 (*NR2F2*) and NUMB Endocytic Adaptor Protein (*NUMB*) in endothelial cells and cardiac cells [16,17]. However, little is known about the role of the NOTCH signaling in avian adipocytes.

*NOTCH1* is well known to regulate various cell fates, including self–renewal, proliferation, and differentiation programs in different cells, such as muscle stem cells [18], primordial germ cells, and spermatogonial stem cells [19]. Conflicting reports exist regarding the role of *NOTCH1* in adipogenic regulation. Ross et al. initially found that decreased expression of *NOTCH1* or inhibition of NOTCH signaling by soluble JAG1 inhibits the differentiation of 3T3–L1 adipocytes [20]. However, subsequent studies argued that *NOTCH1* is dispensable for adipocyte specification and differentiation [21]. More recently, Song et al. reported that blocking *NOTCH1* promotes adipogenic differentiation of mesenchymal stem cells [22]. Given these contradictory findings, investigating the role of *NOTCH1* in avian adipocytes is valuable.

To elucidate the involvement of NOTCH signaling in avian adipocyte differentiation, exploring the effect of *NOTCH1* on gene expression and adipogenesis. The insights gained from this research could lay a solid foundation for constructing a regulatory network of avian adipocyte differentiation and advancing our understanding of poultry fat deposition mechanisms.

## 2. Materials and Methods

### 2.1. Cell Culture

Immortalized chicken preadipocytes 1 (ICP1) was obtained from the Poultry Breeding Lab of Northeast Agricultural University (Supplementary Figure S1A–D) [23], and then cultured in DMEM/F12 (11320033, Gibco, Grand Island, NY, USA) supplemented with 10% FBS (10099141C, Gibco, Grand Island, NY, USA), and 1% penicillin/streptomycin (15140122, Invitrogen, Carlsbad, CA, USA) at 37 °C in a humidified atmosphere with 5% carbon dioxide. Cells from passage three to five were used for the following experiments.

### 2.2. Construction, Amplification, and Purification of Plasmids

For *NOTCH1* intracellular domain (NICD1) overexpression, the coding sequence of NICD (Accession numbers: XM_046901649.1) was determined based on previous studies [24], which is composed of an N–terminal 1–44 amino acids followed by encoding the intracellular domain without C–terminal PEST domain (Accession numbers: XP_046784687.1: 1676–2151 amino acids), which mediates the ubiquitin–mediated degradation of NICD1. The coding sequence of NICD1 was chemically synthesized and then inserted into pcDNA3.1(+) plasmid. For *HES1* overexpression, the coding sequence of *HES1* (Accession numbers: XM_040679737.2) was obtained by PCR amplified from fat cDNA using specific primers (forward: 5′–GACGGCTAGCCACCATGCCTGCCGACCTGA–3′, reverse: 5′–GTCTAGAGGATCCTCACCAGGGCCTCCAGAC–3′), and then inserted into the pcDNA3.1(+) plasmid.

For *PPARγ* promoter reporter plasmid, we amplified the −305 to +104 upstream region of chicken *PPARγ* (Accession numbers: KP736526.1) from chicken genomic DNA using primers (F: 5′-TTCGGTACC GCTCTGTCCTCACAGGAGGT-3′, R: 5′-TTCCTCGAGCTGTCAAGTCTCAGCCGGA-3′), and then cloned into pGL3-Promoter-luc plasmid (Promega, Madison, WI, USA) to generate pGL3-PPARγ-WT, which contained four putative *HES1* binding site (−65/−56: GGCGCGAGCC, −65/−56: GGCTCGCGCC, −282/−273: CGCGCGCGGG, −284/−275: CGCGCGCGAT). Next, we specifically deleted the four putative HES binding sites using DNA synthesis (Tsingke, Beijing, China) to generate pGL3-PPARγ-MUT. Similarly, we also amplified the −1464 to +84 upstream region of chicken *C/EBPα* (Accession numbers: NM_001031459.2) using the primers (F: 5′-TTCGGTACCCCTCCTCTGCAAATGGGTTT-3′, R: 5′- TTCCTCGAGCCGAAGTTCACCATCACGC-3′) to generate pGL3-C/EBPα-WT, which also contained four putative *HES1* binding site (−207/−198: CTCGCGCGTC, −571/−562: GGCACGCTGC, −1293/−1284: AACGCGTGCC, −1293/−1284: GGCACGCGTT). Then, the four putative *HES1* binding site of the plasmid were specifically deleted to generate pGL3-C/EBPα-MUT.

Additionally, the NOTCH luciferase reporter plasmid containing RBP-Jκ binding sites was purchased from Yeasen Biotech (11509ES03, Shanghai, China).

The recombinant plasmids were transformed into *E. coli* DH5α Competent cells (9057, Takara, Kyoto, Japan) and cultured in an LB medium supplemented with 0.1% ampicillin solution overnight (A1170, Solarbio, Beijing, China). The plasmids were isolated and purified from the clone by Endo-Free Plasmid DNA Maxi Kit (19036ES10, Yeasen Biotech, Shanghai, China) and were identified by agarose gel electrophoresis again. All plasmids were validated by sequencing.

### 2.3. RNA Interference

According to the mRNA sequence of chicken *NOTCH1* (Accession numbers: XM_046901649.1), small interfering RNA (siRNA) sequences against *NOTCH1* were purchased from GenePharma (Suzhou, China). The sequences used were as follows: si-NOTCH1, sense 5′-GUGGCAGACUGAUGAAGAATT′-3′ and antisense 5′-UUUGUAGUCAUUGACCCGCTT-3′; si-NC, sense 5′-UUCUCCGAACGUGUCACGUTT-3′ and antisense 5′-ACGUGACACGUUCGGAGAATT-3′.

### 2.4. Transfections

ICP1 cells were seed in 6-well plates with approximately 5 × 10^5^ cells 16 h before transfection. Lipofectamine 3000 and Lipofectamine RNAiMax (L3000150, 13778150, Thermo Fisher Scientific, Waltham, MA, USA) were used for plasmid (3 μg per well) and siRNA (50 pM per well) transfections of ICP1 cells, respectively, following the manufacturer’s instructions. At 72 h after the transfection, cells were processed for adipogenic differentiation, RT-qPCR, and Western blot analysis. Each assay was carried out at least three times independently.

### 2.5. DAPT Treatment

N-[N-(3,5-Difluorophenacetyl-L-alanyl)]-S-phenylglycine-Butyl Ester (DAPT) is a γ-secretase inhibitor that hinders the transactivation of the NOTCH signaling [25]. DAPT (HY-13027, MedChem Express, Monmouth Junction, NJ, USA) was prepared by dissolving in dimethyl sulfoxide (DMSO, HY-10999A, MedChem Express, Monmouth Junction, NJ, USA) and then freshly diluted with culture medium to achieve the desired concentration. The cells were pretreated with either DAPT or DMSO 12 h before the induction of differentiation, Subsequently, throughout the differentiation process, DAPT or DMSO was used in conjunction with 160 μM oleic acid (O1383, Sigma-Aldrich, St. Louis, MO, USA). The ICP1 cells treated with DMSO at 0.05% (*v*/*v*) served as control. ICP1 cells were harvested and processed for Western blot and Oil Red O staining after DAPT treatment for 36 h or 72 h.

### 2.6. Adipogenic Differentiation of ICP1 Cells

The strategy of inducing adipogenic differentiation of ICP1 cells used the same protocol as Shang et al. [26] with minor modifications. Briefly, ICP1 cells were induced to differentiate after 48 h of confluence (0 h) with 160 μM oleic acid in DMEM/F12 supplemented with 10% FBS throughout the differentiation process. At 72 h after the induction, the cells were harvested and used for further assays.

### 2.7. Oil Red O Staining and Quantification

The differentiated cells were fixed with 4% formaldehyde for 10 min. Then, the fixed cells were stained with Oil Red O working solution (G1262, Solarbio, Beijing, China) according to the manufacturer’s manual. Images were captured using an inverted microscope system (Olympus IX53, Tokyo, Japan) at 40× magnification. Lipid contents were quantified by the Oil Red O extraction assay, as described by Ramirez-Zacarias et al. [27]. The BCA Protein Assay Kit (P0010, Beyotime, Shanghai, China) was used to determine the protein concentrations of the cells with identical treatment, which were used for normalized extraction results.

### 2.8. RNA Extraction, cDNA Synthesis, and RT-qPCR Assay

Total RNA was extracted from the differentiated cells using RNAiso reagent (9108, Takara, Kyoto, Japan), and 500 ng of RNA was reverse transcribed into cDNA using PrimeScript RT reagent Kit (RR047A, Takara, Kyoto, Japan). RT-qPCR was performed using SYBR Green PCR Master Mix (4309155, Applied Biosystems, San Francisco, CA, USA) and was conducted using the Bio-Rad CFX96 Touch Real-Time PCR Detection System (Bio-Rad Laboratories, Hercules, CA, USA). Gene-specific primers used in the RT-qPCR are shown in Table 1. The relative gene expression was determined after normalization with housekeeping gene *GAPDH* in the samples using the 2^−ΔΔCt^ method. Each assay was carried out at least three times independently.

### 2.9. Cell Fractionation Assay

The nucleus and cytoplasmic protein of ICP1 cells were collected using a Protein Extraction Kit (P0027, Beyotime, China). Briefly, cells were harvested after tripsinization and washed in ice-cold PBS. Cell pellet was then resuspended with Cytoplasmic protein extraction reagent A for 10 min on ice. Subsequently, Cytoplasmic protein extraction reagent B was added to the reaction mixture for 3 min followed by centrifugation, and the supernatant was stored as cytoplasmic protein. The pellet was resuspended in nuclear protein extraction reagent for 40 min followed by centrifugation, and the supernatant was stored as nucleus protein.

### 2.10. Western Blot Analysis

Cellular proteins were homogenized with RIPA lysis (P0013B, Beyotime, China) supplemented with protease and phosphatase inhibitor cocktails (#P1009; Beyotime, China). The BCA Protein Assay Kit (P0010, Beyotime, China) was used to determine the protein concentrations. Approximately 30 μg of proteins was denatured and subjected to Western blot analysis according to the standard protocols [28]. The immunoreactive proteins were detected on a Chemidoc XRS (Bio-rad Laboratories, Hercules, CA, USA) using BeyoECL Plus kit (P0018S, Beyotime, China) and quantified by Image J (version 1.43) or Imagelab software (version 5.2.1). Antibodies used in the study included the following: Rabbit anti-NOTCH1 (No. 10062-2-AP, Proteintech, Wuhan, China) with 1:500 dilution; Rabbit anti- SOCS3 (No. 14025-1-AP, Proteintech, China) with 1:1000 dilution; Goat anti-NUMB (ab4147, Abcam, Waltham, MA, USA) with 1:1000 dilution; Mouse anti-GAPDH (No.60004-1-Ig, Proteintech, China) with 1:20,000 dilution; Rabbit anti-LMNB1 (No.12987-1-AP, Proteintech, China) with 1:5000 dilution.

### 2.11. Transcription Factor Binding Sites Prediction

The JASPAR CORE vertebrate database [29] was utilized to identify and examine potential transcription factor binding sites in the 2000 bp upstream region of *PPARγ* and *C/EBPα* (Accession numbers: KP736526.1 and NM_001031459.2). The threshold for profile score relevance was established at 85% for this analysis.

### 2.12. Dual-Luciferase Reporter Assay

To examine the response of NICD1 to NOTCH signaling, ICP1 cells were co-transfected with pcDNA3.1-NICD1 or pcDNA3.1 and NOTCH luciferase reporter plasmid, along with pRL-TK plasmid (Promega; ratio 24:24:1). To analyze the effect of *HES1* on the reporter gene activity of the *PPARγ* or *C/EBPα* upstream region, cells were co-transfected with pcDNA3.1-HES1 or pcDNA3.1(+) and pGL3-PPARγ-WT or pGL3-PPARγ-MUT (pGL3-C/EBPα-WT or pGL3-C/EBPα-MUT), along with pRL-TK (ratio 24:24:1). At 72 h after the transfection, the cells were harvested, lysed, and assessed with a dual luciferase reporter kit (E2920, Promega). The relative Luciferase activity of each construct was measured by Infinate^®^ M200 PRO microplate reader (Tecan, Morrisville, NC, USA) and was calculated as the Firefly/Renilla luciferase activity ratio. Each assay was independently conducted at least three times.

### 2.13. RNA-seq Analysis

Two previously published mRNA-seq datasets were downloaded from NCBI (Accession number: SRX4646736, PRJNA1069883) [9,10] to analyze the expression patterns of NOTCH signaling-related genes during the differentiation process of chicken abdominal or duck subcutaneous adipocytes, including two NOTCH receptors (*NOTCH1*, *NOTCH2*), six NOTCH ligand genes (*DLL1*, *DLL4*, *JAG1*, *JAG2*, *DLK1*, and *DLK2*), and three NOTCH-dependent transcription factors (*HES1*, *HEY1*, and *HEY2*). Trimmed reads were aligned against GRCg7b or CAU_wild_1.0 using HISAT2 [30]. The aligned files were passed into HTSeq [31] for gene quantification into TPM units. DESeq2 (v.1.28.1) [32] package was used to normalize and perform differential expression analysis on the quantified reads.

### 2.14. Statistical Analysis

All replicate experiments were biological replicates. Non-parametric statistical analysis was conducted using GraphPad Prism 9. Results are shown as mean ± standard deviation. Unpaired Student’s *t*-test was employed for comparison between two groups, and One-way ANOVA followed by the Tukey test was utilized for comparison among more than two groups. All experiments yielded statistically significant results, with * indicating a significant difference (*p* < 0.05), and ** indicating a very significant difference (*p* < 0.01).

## 3. Results

### 3.1. NOTCH1 Expression during Avian Adipocyte Differentiation

**Figure 1:**
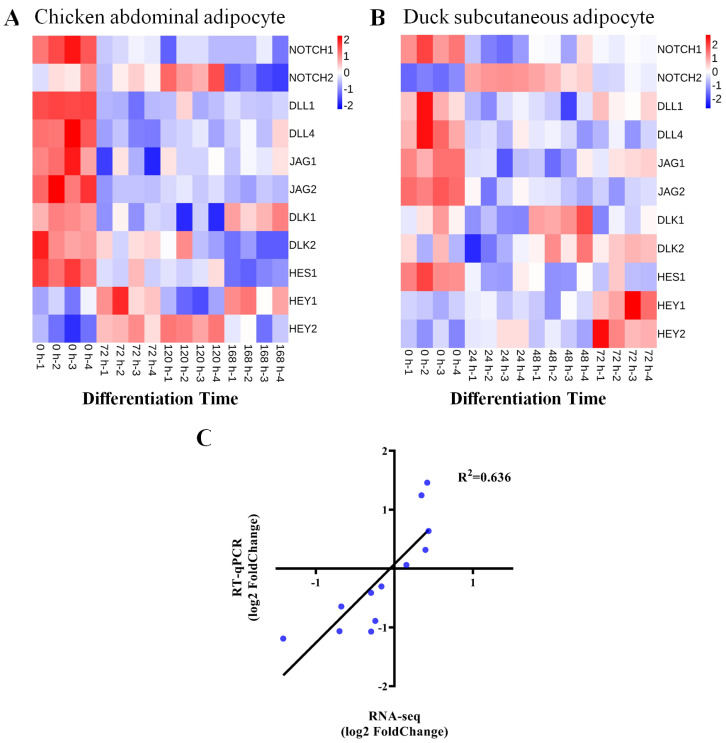
The expression changes in NOTCH signaling during avian adipocyte differentiation. (**A**) Heatmap analysis of expression patterns for NOTCH signaling–related genes identified with mRNA–seq analysis of gene expression data from chicken abdominal adipocytes at 0 h, 72 h, 120 h, 168 h post induction (*n* = 4, Raw in Z–Score). (**B**) Heatmap analysis of expression patterns for NOTCH signaling–related genes identified with mRNA–seq analysis of gene expression data from Pekin duck subcutaneous adipocytes at 0 h, 24 h, 48 h, 72 h post induction (*n* = 4, Raw in Z–Score). (**C**) Comparison between mRNA–seq and RT–qPCR expression measurements for the chosen genes. The graph illustrates the log2 fold changes for both mRNA–seq and RT–qPCR data.

To further investigate the role of the NOTCH signaling in avian adipocytes, we profiled the expression of several signaling members during the differentiation process of chicken abdominal adipocyte line (ICP1 cells) and primary duck subcutaneous adipocyte using two previously published mRNA-seq datasets [9,10]. Interestingly, NOTCH signaling members showed similar expression patterns in both avian species (Figure 1A,B). The expression of *NOTCH1* and its ligands *DLL1*, *DLL4*, *DLK1*, *JAG1*, and *JAG2* is significantly decreased during adipocyte differentiation, whereas *NOTCH2* shows the opposite expression patterns. Meanwhile, we analyzed the expressions of the downstream transcription factors of NOTCH signaling, including *HES1*, *HEY1*, and *HEY2*. It was clear that the expression of *HEY1* and *HEY2* were significantly increased after induction of adipogenic differentiation, but the expression of *HES1* was significantly decreased (Figure 1A,B). To validate the results of mRNA-seq analysis results, we used RT-qPCR in ICP1 cells to detect the expression of four genes (*NOTCH1*, *HES1*, *HEY1*, and *HEY2*) at the same differentiation time point. The results showed a good correlation between the fold change measured by mRNA-seq and RT-qPCR (R^2^ = 0.636; Figure 1C).

**Figure 2:**
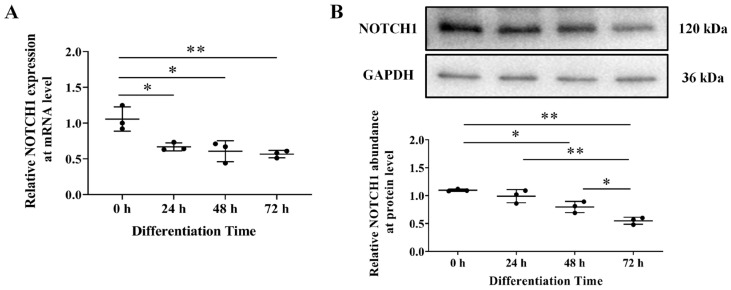
*NOTCH1* is inhibited during adipocyte differentiation. (**A**) ICP1 cells were induced to differentiate with 160 μM oleic acid and total RNA was extracted at specified time points (0 h corresponds to the time of oleic acid supplementation). Subsequently, RT−qPCR was conducted to assess *NOTCH1* expression throughout adipocyte differentiation (*n* = 3). The mRNA expression was normalized to GAPDH mRNA, and the results are presented relative to the mRNA level of *NOTCH1* at 0 h, which is set as 1. (**B**) Western blot analysis of protein abundance of *NOTCH1* during the ICP1 cells differentiation; GAPDH was utilized as an internal control. The protein abundance of *NOTCH1* and *GAPDH* were quantitatively compared (*n* = 3). The data are presented as mean ± SD, and statistical significance was determined through one−way ANOVA. * *p* < 0.05; ** *p* < 0.01.

The endogenous expression of *NOTCH1* was also evaluated by RT–qPCR and Western blot during ICP1 differentiation. We found that *NOTCH1* is inhibited during adipocyte differentiation at both the mRNA and protein levels (*p* < 0.05; Figure 2A,B).

### 3.2. NOTCH1 Regulates Expression of Signaling-Related Genes

**Figure 3:**
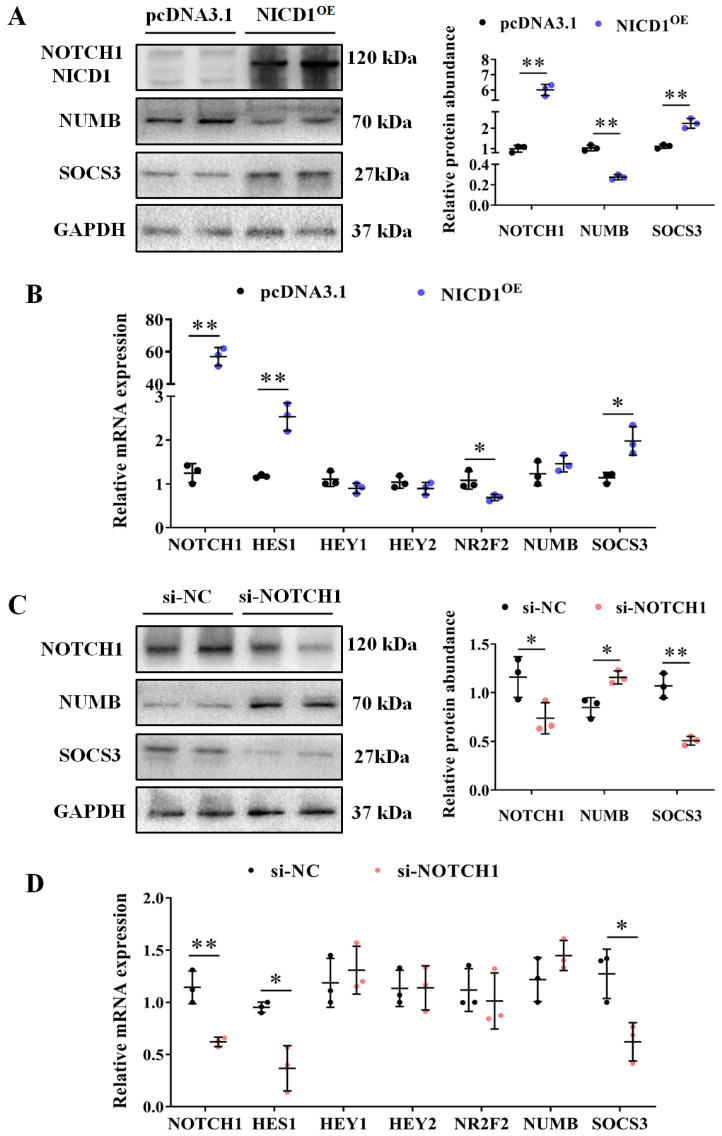
Effect of *NOTCH1* on signaling−related genes in ICP1 cells. (**A**) Western blot analysis of protein abundance of *NOTCH1*, *NUMB*, *SOCS3*, and *GAPDH* in NICD1 overexpression ICP1 cells and control cells; GAPDH is an internal control. The protein abundance of NOTCH1, NUMB, SOCS3, and GAPDH was quantitatively compared (*n* = 3). (**B**) The relative mRNA expression of *NOTCH1*, *HES1*, *HEY1*, *HEY2*, *NR2F2*, *NUMB*, and *SOCS3* in NICD1 overexpression ICP1 cells and control cells was determined using RT−qPCR; *GAPDH* served as an internal control (*n* = 3). (**C**) Western blot analysis of protein abundance of *NOTCH1*, *NUMB*, *SOCS3*, and *GAPDH* in *NOTCH1* knockdown ICP1 cells and control cells. The abundance of each protein was quantitatively compared (*n* = 3). (**D**) Relative mRNA expression of *NOTCH1*, *HES1*, *HEY1*, *HEY2*, *NR2F2*, *NUMB*, and *SOCS3* in *NOTCH1* knockdown ICP1 cells and control cells was determined using RT−qPCR (*n* = 3). Data are shown as mean ± SD, and significance was determined using Student’s *t*−tests. * *p* < 0.05; ** *p* < 0.01.

To investigate the effect of *NOTCH1* in avian adipocytes, we generated an ICP1 line with endothelial-specific NOTCH intracellular domain (NICD1) overexpression as the constitutively active form of *NOTCH1*. As expected, NICD1 overexpression induces promoter activity dependent on the RBP–Jκ site (*p* < 0.01; Supplementary Figure S2). Subsequently, we investigated the expression of several of its targets, including *SOCS3*, *NR2F2*, *NUMB*, etc. The immunoblotting results showed that the protein abundance of *NOTCH1* and *SOCS3* were increased in NICD1–overexpressing cells, while *NUMB*, an adaptor protein that promotes *NOTCH1* degradation by recruiting the E3 ubiquitin ligase, was decreased (*p* < 0.05; Figure 3A). In addition, the mRNA levels of downstream genes were quantified by RT–qPCR. The mRNA levels of *HES1* and *SOCS3* were increased, as well as decreased *NR2F2* in NICD1–overexpressing cells (*p* < 0.05; Figure 3B). Surprisingly, the mRNA level of *NUMB* was not affected by NICD1 overexpression, implying posttranscriptional regulation of *NUMB* by *NOTCH1*. Additionally, the mRNA levels of *HEY1* and *HEY2* were not affected by NICD1 (Figure 3B).

To further elucidate the regulation of endogenous *NOTCH1* in avian adipocytes, we employed siRNA–NOTCH1 to inhibit its expression in cells. The efficiency of the siRNA–mediated *NOTCH1* knockdown was validated by RT–qPCR and Western blot (Figure 3C,D). Contrary to the observations in NICD1–overexpressing cells, the downstream genes *HES1* and *SOCS3* were down–regulated, while *NUMB* was up–regulated in the *NOTCH1* knockdown cells, suggesting that these genes are regulated by *NOTCH1* in avian adipocytes (*p* < 0.05; Figure 3C,D).

### 3.3. NOTCH1 Inhibits Avian Adipocyte Differentiation

**Figure 4:**
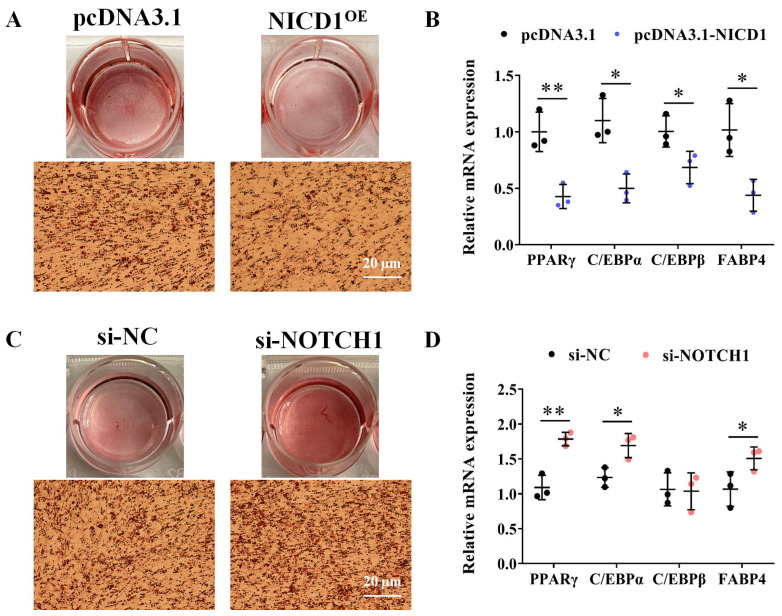
*NOTCH1* inhibits fat accumulation in differentiating adipocyte. (**A**) Cultured ICP1 cells expressing pcDNA3.1 or pcDNA–NICD1 was induced to differentiate into adipocytes with 160 μM oleic acid. At the end of induction (72 h), cells were subjected to Oil Red O staining to evaluate lipid accumulation. All micrographs were captured at the same magnification, and a 20 μm scale bar is provided in the lower right panel for reference. (**B**) Relative mRNA expression of mature adipocyte marker genes in NICD1 overexpression ICP1 cells and control cells was determined using RT–qPCR; *GAPDH* served as an internal control (*n* = 3). (**C**) Representative images of *NOTCH1* knockdown promoted the lipid accumulation by ORO staining on 72 h post induction. (**D**) Relative mRNA expression of mature adipocyte marker genes in *NOTCH1* knockdown ICP1 cells and control cells was determined using RT–qPCR (*n* = 3). Data are shown as mean ± SD and significance was determined using Student’s *t*–tests. * *p* < 0.05; ** *p* < 0.01.

To specifically assess the potential role of *NOTCH1* in adipogenesis, we examined the effects of overexpression of NICD1 on adipogenic differentiation of ICP1 cells. The results indicated that NICD1 overexpression inhibits oleic acid–induced adipogenesis, as shown by Oil Red O (ORO) staining of neutral lipids (*p* < 0.01; Figures 4A and  S3A), and reduces mRNA levels of the pro−adipogenic factors *PPARγ*, *C/EBPα*, *C/EBPβ*, and *FABP4* (*p* < 0.05; Figure 4B), whereas knockdown of *NOTCH1* in ICP1 cells exhibited enhanced differentiation into adipocytes, as evidenced by increases in ORO staining, along with elevated mRNA levels of the adipocyte markers *PPARγ* and *C/EBPα* (*p* < 0.05; Figures 4C,D and  S3B).

### 3.4. DAPT, an Inhibitor of NOTCH Signaling, Promotes Adipocyte Differentiation

**Figure 5:**
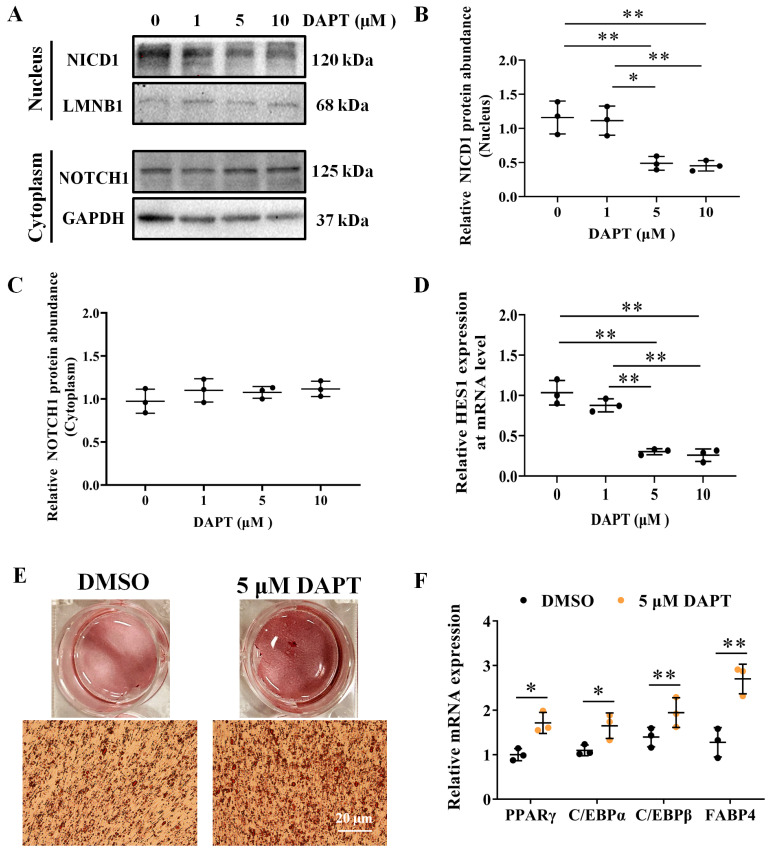
DAPT inhibits *NOTCH1* and *HES1* and promotes adipogenic differentiation. (**A**) Western blot analysis of protein abundance of NICD1 (nuclear), LMNB1 (nuclear), NOTCH1 (cytoplasm), and GAPDH (cytoplasm) in ICP1 cells treated with various concentrations of DAPT for 48 h; LMNB1 and GAPDH as internal control of nuclear protein and cytoplasmic protein, respectively (*n* = 3). (**B**) The protein abundance of NICD1 (nuclear) and LMNB1 (nuclear) was quantitatively compared (*n* = 3). (**C**) The protein abundance of *NOTCH1* (cytoplasm) and *GAPDH* (cytoplasm) was quantitatively compared (*n* = 3). (**D**) The mRNA level of *HES1* in ICP1 cells treated with various concentrations of DAPT for 48 h was determined using RT-qPCR (*n* = 3). (**E**) Representative images of 5 μM DAPT promoted the lipid droplet formation in ICP1 cells by ORO staining on 72 h post induction. All micrographs are the same magnification, and a 20 μm scale bar is provided in the lower right panel for reference. (**F**) Relative mRNA expression of mature adipocyte marker genes in 5 μM DAPT treated with ICP1 cells and alone DMSO treated with cells on 72 h post induction was determined using RT–qPCR (*n* = 3). Data are shown as mean ± SD and significance was determined using One–way ANOVA or Student’s *t*–tests. * *p* < 0.05; ** *p* < 0.01.

We also employed DAPT, a γ–Secretase inhibitor, to block NOTCH cleavage and activation. ICP1 cells were treated with DAPT at 0, 1, 5, and 10 μM for 48 h, and the nuclear protein abundance of NICD1 (Figure 5A–C) and mRNA expression of *HES1* was detected (Figure 5D). As expected, incubation of the cells with DAPT at 5 and 10 μM significantly decreased the nuclear protein abundance of NICD1 (*p* < 0.05; Figure 5B) and *HES1* mRNA expression (*p* < 0.01; Figure 5D). Subsequently, ICP1 cells were treated with DAPT at 5 μM or DMSO (0.05% *v*/*v*) at 12 h before differentiation. Following adipogenic induction, significantly increased ORO staining (*p* < 0.01; Figures 5E and  S4) and mRNA levels of *PPARγ*, *C/EBPs*, and *FABP4* were observed in DAPT treated cells compared to cells treated with DMSO (*p* < 0.05; Figure 5F). These findings demonstrate the crucial role of NOTCH signaling in adipogenesis and *NOTCH1* inhibits avian adipocyte differentiation.

### 3.5. HES1 Inhibits the Expression of PPARγ and C/EBPα by Directly Binding to Their Upstream Region

**Figure 6:**
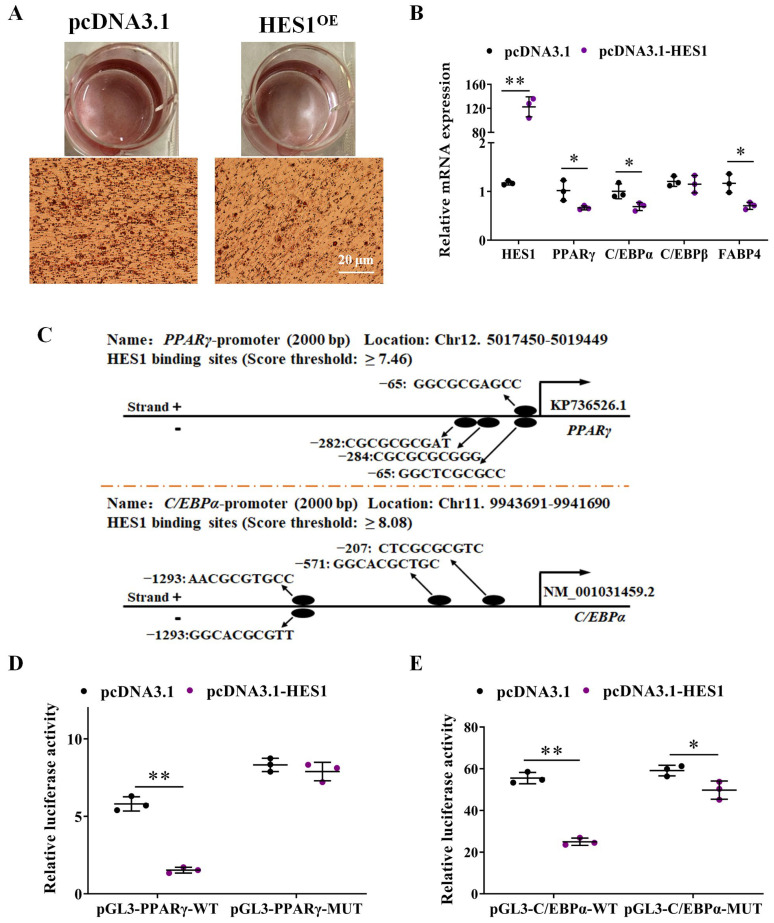
*HES1* overexpression inhibits *PPARγ* and *C/EBPα* and promotes adipogenic differentiation. (**A**) Cultured ICP1 cells expressing pcDNA3.1 or pcDNA–HES1 was induced to differentiate into adipocytes with 160 μM oleic acid. At the end of differentiation (72 h), cells were subjected to Oil Red O staining to evaluate lipid accumulation. All micrographs were captured at the same magnification, and a 20 μm scale bar is provided in the lower right panel for reference. (**B**) Relative mRNA expression of *HES1* and mature adipocyte marker genes in *HES1* overexpression ICP1 cells and control cells was determined using RT–qPCR; *GAPDH* served as an internal control (*n* = 3). (**C**) Predicted binding sites and putative binding sequence for the *HES1* transcription factor in the *PPARγ* and *C/EBPα* promoter as identified in the JASPAR database. (**D**) The cells were co–transfected with pGL3–PPARγ–WT or pGL3–PPARγ–MUT and pcDNA3.1–HES1 or pcDNA3.1, and pRL–TK plasmid (24:24:1). After 72 h of co–transfection, relative luciferase activity was measured. The effect of *HES1* overexpression on the luciferase activity of pGL3–PPARγ–WT and pGL3–PPARγ–MUT in ICP1 cells (*n* = 3). (**E**) The effect of *HES1* overexpression on the luciferase activity of wild type reporter pGL3–*C/EBPα*–WT and mutation reporter pGL3–*C/EBPα*–MUT in ICP1 cells (*n* = 3). Data are shown as mean ± SD and significance was determined using Student’s *t*–tests. * *p* < 0.05; ** *p* < 0.01.

Furthermore, we also investigated the effect of *HES1*, a NOTCH downstream active transcription factor, on avian adipogenesis via a gain-of-function study. In comparison to the control group, *HES1*–overexpressing ICP1 cells exhibited an approximately 120-fold increase in *HES1* mRNA level, as revealed by RT–qPCR analysis. This surge in *HES1* levels corresponded to a significant reduction in ORO staining and a decrease in mRNA levels of adipogenic markers, including *PPARγ*, *C/EBPα*, and *FABP4* (*p* < 0.05; Figures 6A,B and  S5). To strengthen the evidence supporting the direct regulatory connection between *HES1* and the adipogenic transcription factor, we employed the JASPAR prediction database. Our analysis revealed multiple potential binding sites for the HES1 transcriptional repressor in the upstream region of *PPARγ* and *C/EBPα* (Figure 6C).

To test whether *HES1* directly regulates *PPARγ* and *C/EBPα* expression via binding to the upstream region, we cloned the 409 bp upstream promoter of *PPARγ* and the 1548 bp upstream promoter of *C/EBPα* into the pGL3–Promoter plasmid (pGL3–PPARγ–WT and pGL3–C/EBPα–WT), each of which contained four potential binding sites for HES1. After deleting the four binding sites, two corresponding mutant promoter reporter plasmids were constructed (pGL3–PPARγ–MUT and pGL3–C/EBPα–MUT). The results showed that transfection of pcDNA3.1–HES1 significantly inhibited the wild–type reporters’ activity pGL3–PPARγ–WT and pGL3–C/EBPα–WT compared with control pcDNA3.1 (*p* < 0.05; Figure 6D,E). On the contrary, the reporter activity of the mutation–type reporter GL3–PPARγ–MUT and pGL3–C/EBPα–MUT were not affected by intracellular *HES1* overexpression ((*p* > 0.05; Figure 6D,E). These results demonstrate the important role of the *NOTCH1*/*HES1* axis in effectively inhibiting avian adipocyte differentiation, thereby presenting a potential target for regulating fat deposition in avian species.

## 4. Discussion

Excessive fat deposition has a profound impact on both the efficiency and quality of meat production [33]. Fat deposition can be driven either by hyperplasia or hypertrophy, a process known as adipogenesis at the cellular level [4]. Despite substantial research employing various strategies such as genetic selection, feeding supplementation, and multi−omics research to understand the genetic regulation of adipogenesis, the molecular mechanisms underlying avian fat deposition remain largely unknown.

*NOTCH1* is a conserved receptor of communication between adjacent cells and broadly involved in cellular differentiation, proliferation, and apoptosis throughout all development stage [11]. Our prior and ongoing research has revealed that the expression of several genes related to the NOTCH signaling pathway undergoes alterations during avian adipocyte differentiation. These findings strongly suggest that these genes may have a pivotal role in regulating avian adipogenesis. However, it remains unclear whether *NOTCH1*’s role is pro− or anti−adipogenesis. In line with the findings of Song et al. [22] and Wang et al. [34], our findings suggest that *NOTCH1* negatively regulates avian adipocyte differentiation.

Our study also revealed a significant association between the mRNA levels of *HES1* and *NOTCH1* activity, with *HES1* expression decreasing following adipogenic differentiation. The functional role of *HES1* in adipogenesis was further confirmed through a gain−of−function study in ICP1 cells, resulting in impaired adipogenesis, as evidenced by a decrease in intracellular lipid droplet accumulation. In contrast, previous studies by Ross et al. demonstrated that *HES1* is required for 3T3−L1 differentiation by suppressing *DLK*/*PREF1* [20]. Meanwhile, Lei et al. reported that *HES1* inhibits adipogenesis of porcine mesenchymal stem cells via transcriptional repression of *FAD24* [35]. Moreover, we confirmed that *HES1* inhibited the expression and promoter activity of *PPARγ* and *C/EBPα* in avian adipocyte by luciferase assay and gene expression analysis.

Chicken *PPARγ* gene is characterized by five transcript variants (*cPPARγ1*–*5*) generated by alternative promoter usage and alternate splicing, resulting in two protein isoforms (PPARγ1 and PPARγ2) [36]. PPARγ2 protein isoform is distinguished by an extra six amino acids at the N—terminal, which include the activation function—1 domain, compared to PPARγ1 protein isoform [36]. The differences between the two chicken PPARγ protein isoforms in expression patterns, proliferation and differentiation have been well characterized in several studies [6,37]. Given the notably higher expression of *cPPARγ1* transcript (encoding PPARγ1 protein isoform) in chicken adipocytes and tissues compared to other transcript variants, our study primarily analyzed the effect of *HES1* on the transcriptional activity of the promoter of *cPPARγ1* transcript. Additionally, bioinformatics analysis identified several putative *HES1* binding sites that existed in the promoter of *cPPARγ2* transcript (encoding PPARγ2 protein isoform; Supplementary Table S1), suggesting that chicken PPARγ2 protein isoform may also be regulated by *HES1*.

Furthermore, increases in *HEY1*, *HEY2*, and *SOCS3*, as well as decreases in *NUMB* and *NR2F2* were detected in the NICD1–overexpressing cells relative to controls, as determined by RT–qPCR or Western blot, implying these genes may be involved in the regulation of avian adipogenesis through *NOTCH1*. Previous literature has suggested that *NR2F2* directly binds to the promoter regions of *HEY1* and *HEY2*, leading to transcriptional repression [38]. Intriguingly, our separate study has reported that *NR2F2* is an anti-adipogenic factor in avian adipocytes and its expression also decreased post induction [39]. However, regulatory connection between *NOTCH1* and *NR2F2* in avian needs additional exploration. The precise understanding of the cascade of these molecular events and the way to regulate them will certainly be crucial to efficiently fight fat deposition.

One limitation of our study is the use of immortalized chicken abdominal adipocytes, ICP1, as an in vitro avian cell culture model for the analyses of the effects of *NOTCH1* on adipocyte differentiation and gene expression. However, different depots of fat, such as abdominal fat, subcutaneous fat, and intermuscular fat, differ in their developmental origin, anatomical location, and response to metabolic stimuli [40,41]. Moreover, the adipocytes derived from different depots have shown that they functionally differ in lipolysis, adipogenic potential, and thermogenic activity [42,43]. Therefore, it would be intriguing to reproduce our results in distinct cell subsets from other depots or more detailed functional analyses. Additionally, it is worth investigating the in vivo functions of *NOTCH1* in chicken adipogenesis and fat development using different gene editing strategies.

## 5. Conclusions

In summary, this study not only reaffirms the significance of NOTCH signaling in regulating avian adipogenesis, but also furnishes comprehensive evidence supporting *NOTCH1*’s role as a repressor of adipocyte differentiation in the avian adipocyte model. Given the pivotal role of adipocyte differentiation in adipose tissue development and function, elucidating the mechanism by which NOTCH signaling modulates adipogenesis becomes imperative. Such understanding is crucial for enabling more efficient selection methods aimed at mitigating excessive fat deposition and thereby enhancing both chicken production efficiency and meat quality.

## Figures and Tables

**Table 1:** Sequences of primers used for RT-qPCR.

Gene Symbol	Accession Numbers	Sequence (5′–3′)	Length (bp)
*NOTCH1*	XM_046928731.1	F: AACGCTGTGGATGATCTGGG	131
		R: AGCGGGGTCTCCTCCTTATT	
*PPARγ*	NM_001001460	F: GTGCAATCAAAATGGAGCC	170
		R: CTTACAACCTTCACATGCAT	
*FABP4*	NM_204290	F: ATGTGCGACCAGTTTGT	143
		R: TCACCATTGATGCTGATAG	
*C/EBPα*	NM_001031459	F: GGAGCAAGCCAACTTCTACG	181
		R: GTCGATGGAGTGCTCGTTCT	
*C/EBPβ*	NM_205253	F: CGCCCGCCTTTAAATCCATG	151
		R: GGGCTGAAGTCAATGGCTCT	
*HES1*	XM_040679737	F: GGACGCGCTGAAGAAGGATA	203
		R: CTTCGCAGGTGGAGAGGAAC	
*HEY1*	XM_040665051	F: TGGCTGAAGTGGCTCGATAC	175
		R: TGAGGGTGATGTCCAAAGGC	
*HEY2*	XM_040669098	F: GGGCAGCGAGAACAACTACT	125
		R: CCCGGCGCCTTTTCTCTATA	
*SOCS3*	NM_204600.2	F: GCCTCAAGACGTTCAGCTCT	196
		R: GTCTTGACGCTGAGGGTGAA	
*NUMB*	AF176086	F: ACAAAACCCGTGACAGTGGT	168
		R: GGCACGGACAGTCTTTGAGA	
*NR2F2*	XM_046924463	F: TGACCTGGAGCGAGTTGTTC	185
		R: GTCGACATGCAACGCTTTCA	
*GAPDH*	NM_204305	F: CCTCTCTGGCAAAGTCCAAG	200
		R: CATCTGCCCATTTGATGTTG	

## Data Availability

The data presented in this study are available on request from the corresponding author. The mRNA−seq datasets used in this study were taken from previously published paper and is available in NCBI (Accession number: SRX4646736, PRJNA1069883).

## Supplementary Materials

The following supporting information can be downloaded at: https://www.mdpi.com/article/10.3390/ani14040585/s1. Figure S1: Representative phase contrast images of ICP1 cells approximately 24 h after seeding; Figure S2: NICD1 promoted the relative luciferase activity dependent on RBP−Jκ site; Figure S3: Quantification of ORO staining after *NOTCH1* overexpression or knockdown or control; Figure S4: Quantification of ORO staining after DAPT or DMSO treatment; Figure S5: Quantification of ORO staining after *HES1* overexpression or control. Table S1: The putative binding sites of transcription factor *HES1* within 2000 bp upstream region of *cPPARγ2* transcript (Chr12: 5018144− 5020143). The original images of staining cells. The original Western blot figures.
