# Supplementary material for: NOTCH1 as a Negative Regulator of Avian Adipocyte Differentiation: Implications for Fat Deposition
Source: Animals (Basel). 2024 Feb 9;14(4):585. doi: 10.3390/ani14040585 (PMC10886207; doi:10.3390/ani14040585)
Supplement: Supplementary file 1 [file animals-14-00585-s001.zip › animals-2841143-supplementary.pdf]

Supplementary Material

NOTCH1 as a negative regulator of avian adipocyte differentiation: implications for fat disposition

Zheng Wang[1†], Yue Su[1†], Mingyu Zhao[1], Zhenhua Ma[1], Jianhui Li[2], Zhuocheng Hou[3], Huifeng Li[1]*

*** Correspondence:** Huifeng Li, lhftom@sxau.edu.cn

1 Supplementary Figures

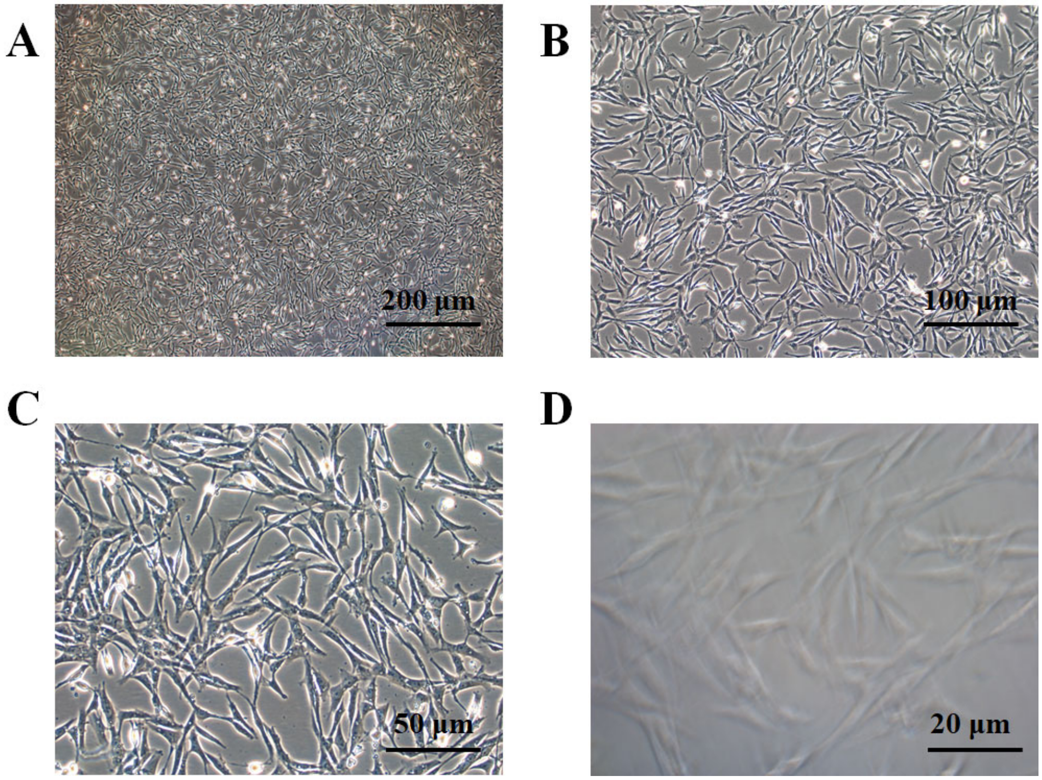

Figure S1. Representative phase contrast image of ICP1 cells approximately 24 h after seeding. (A) at 4× magnification objective. (B) at 10× magnification objective. (A) at 20× magnification objective. (B) at 40× magnification objective.

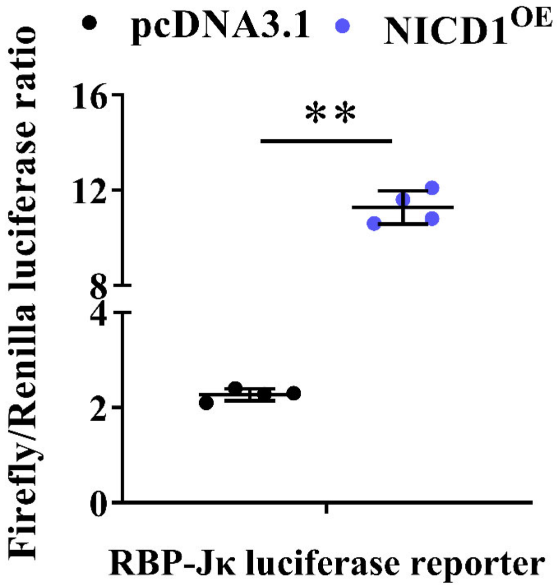

Figure S2. NICD1 promoted the relative luciferase activity dependent on RBP-Jκ site (n=4). Data are shown as mean ± SD and significance was determined using Student's *t-tests*. $^{**}P < 0.01$.

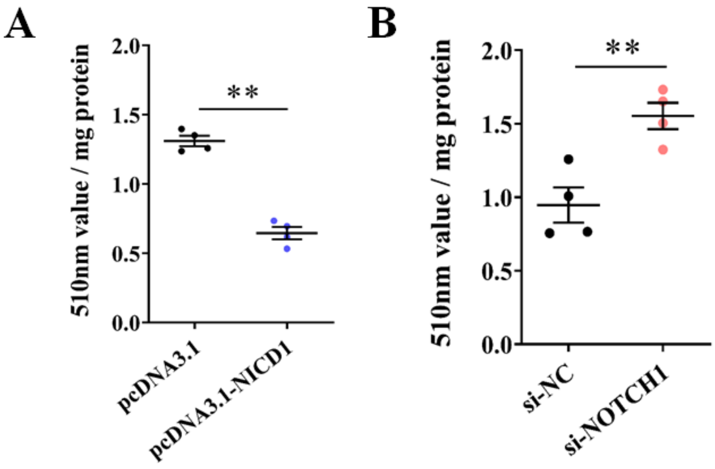

Figure S3. Quantification of Oil-Red-O staining after *NOTCH1* overexpression or knockdown or control. (A) Comparison of the lipid droplet content in NICD1 overexpression ICP1 cells and control cells obtained by oil red O staining and

extraction methods (n=4). (B) Comparison of the lipid droplet content in *NOTCH1* knockdown ICP1 cells and control cells (n=4). Data are shown as mean ± SD and significance was determined using Student's *t-tests*. $^{**}P < 0.01$.

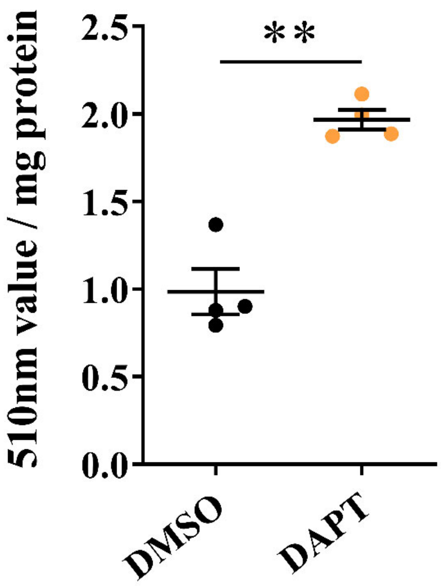

Figure S4. Quantification of Oil-Red-O staining after DAPT or DMSO treatment (n=4). Data are shown as mean ± SD and significance was determined using Student's *t-tests*. $^{**}P < 0.\ 01$.

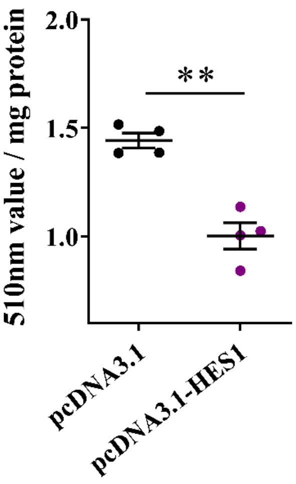

Figure S5. Quantification of Oil-Red-O staining after *HES1* overexpression or control. (n=4). Data are shown as mean ± SD and significance was determined using Student's *t-tests*. **$P < 0.01$.

Table S1. The putative binding sites of transcription factor *HES1* within 2000 bp upstream region of *cPPARγ2* transcript (Chr12: 5018144- 5020143).

Putative binding sites	Start	End	Strand	Score
GGCGCGTG	5020107	5020114	+	12.92
GGCGCGTGAA	5020107	5020116	+	9.77
GGCGTGTG	5019635	5019642	+	8.44
CGCGCGCG	5019168	5019175	+	8.35
CGCGCGCG	5019168	5019175	-	8.35
TTCACGCGCC	5020107	5020116	-	8.92
GGCGTGTGGG	5019635	5019644	+	8.87
CGCGCGCGGG	5019166	5019175	-	8.77
TTCACGCG	5020109	5020116	-	7.47
GGCGCGAGCC	5019385	5019394	+	8.37

2 The original images of staining cells:

Figure 3A

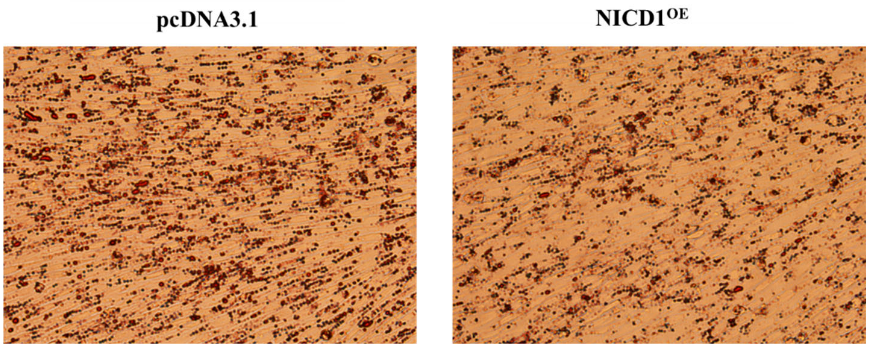

Figure 3C

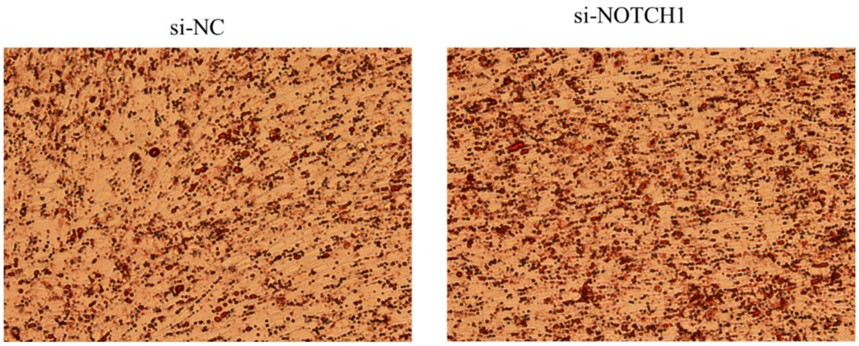

Figure 4E

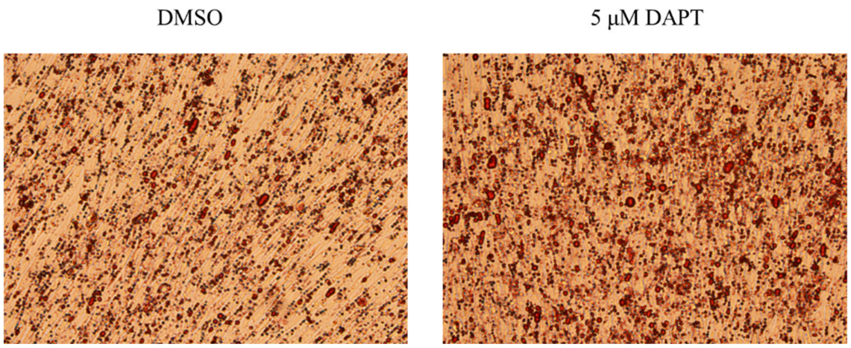

Figure 5A

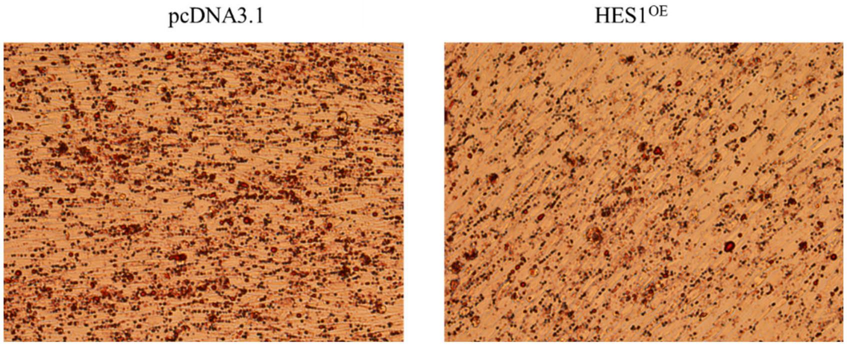

3 The original Western blot figures:

Figure 1B

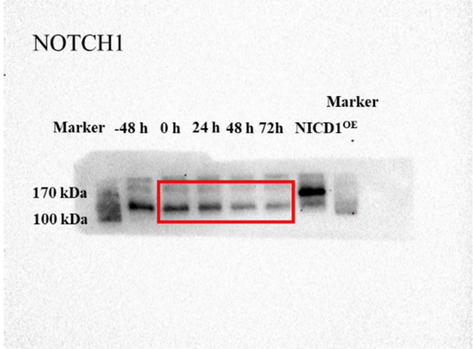

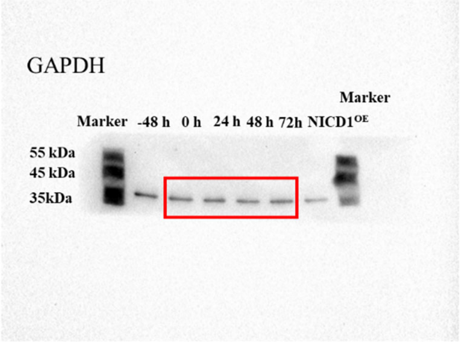

Figure 2A

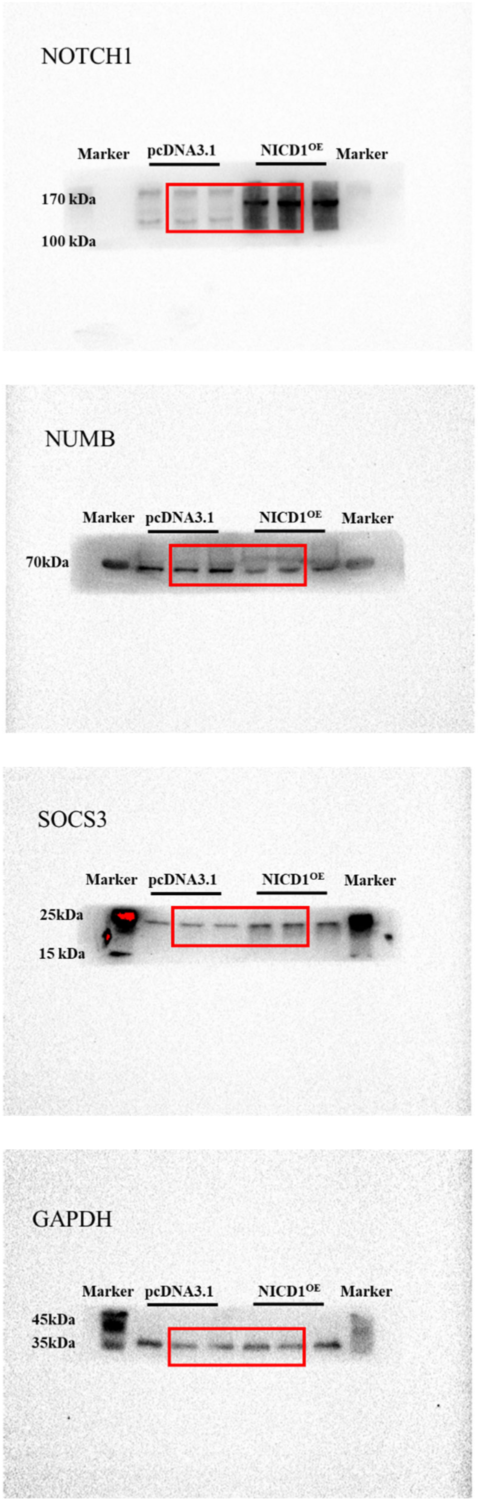
NOTCH1
Marker
pcDNA3.1
NICD1OE
Marker
170 kDa
100 kDa
NUMB
Marker
pcDNA3.1
NICD1OE
Marker
70kDa
SOCS3
Marker
pcDNA3.1
NICD1OE
Marker
25kDa
15 kDa
GAPDH
Marker
pcDNA3.1
NICD1OE
Marker
45kDa
35kDa

Figure 2C

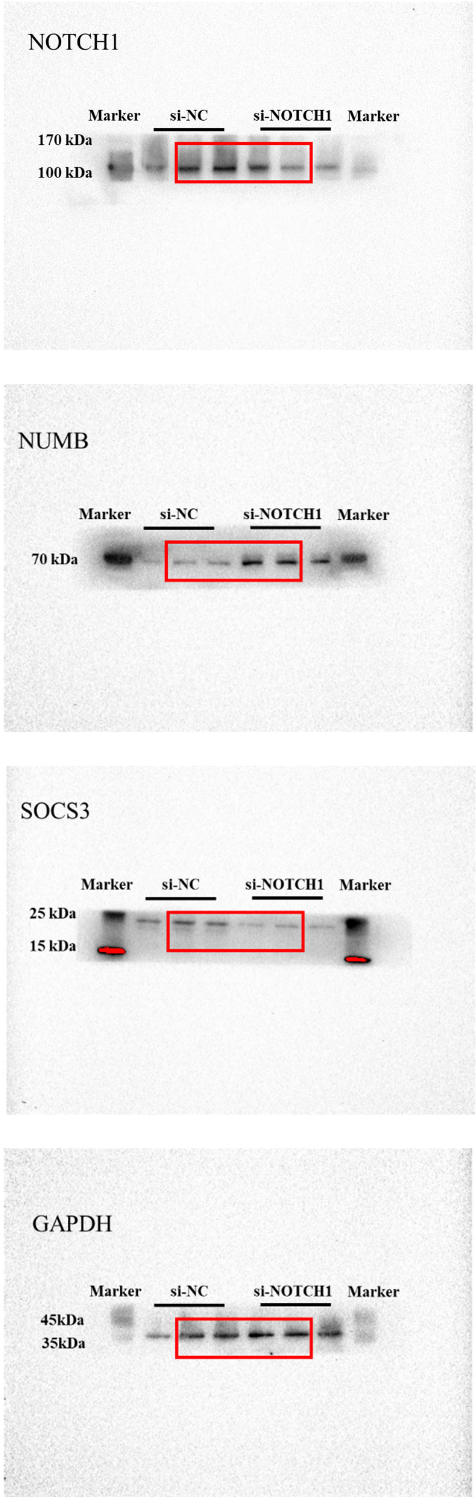
NOTCH1
Marker
si-NC
si-NOTCH1
Marker
170 kDa
100 kDa
NUMB
Marker
si-NC
si-NOTCH1
Marker
70 kDa
SOCS3
Marker
si-NC
si-NOTCH1
Marker
25 kDa
15 kDa
GAPDH
Marker
si-NC
si-NOTCH1
Marker
45kDa
35kDa

Figure 4A

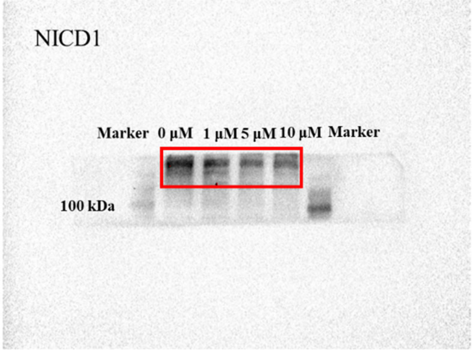
NICD1
Marker 0 μM 1 μM 5 μM 10 μM Marker
100 kDa

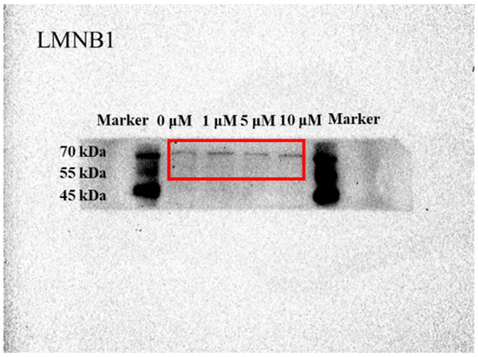
LMNB1
Marker 0 μM 1 μM 5 μM 10 μM Marker
70 kDa
55 kDa
45 kDa

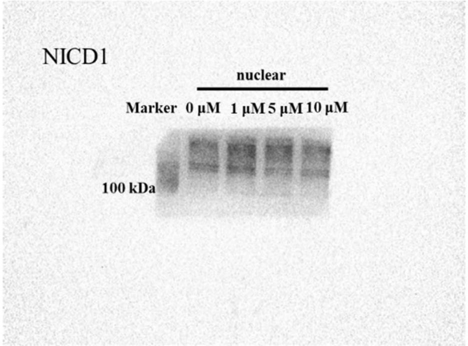
NICD1
nuclear
Marker 0 μM 1 μM 5 μM 10 μM
100 kDa

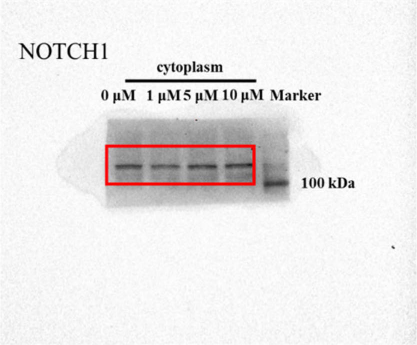
NOTCH1
cytoplasm
0 μM 1 μM 5 μM 10 μM Marker
100 kDa

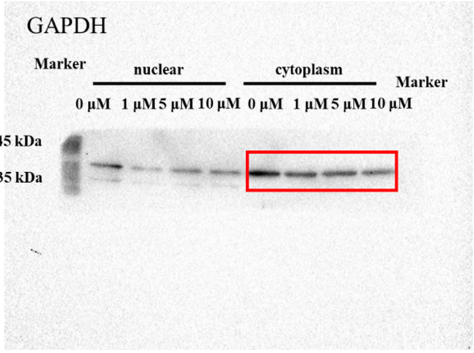
GAPDH
Marker
nuclear
cytoplasm
Marker
0 μM 1 μM 5 μM 10 μM 0 μM 1 μM 5 μM 10 μM
45 kDa
35 kDa
